# Supplementary material for: Antioxidant and Leishmanicidal Evaluation of Pulicaria Inuloides Root Extracts: A Bioguided Fractionation
Source: Pathogens. 2019 Oct 23;8(4):201. doi: 10.3390/pathogens8040201 (PMC6963631; doi:10.3390/pathogens8040201)
Supplement: Supplementary file 1 [file pathogens-08-00201-s001.pdf]

Electronic Supporting Information

Antioxidant and leishmanicidal evaluation of *Pulicaria inuloides* root extracts: a bioguided fractionation

Hamza Fadel[1], Ines Sifaoui[2,3], Atteneri López-Arencibia[2], María Reyes-Batlle[2], Ignacio A. Jiménez[4], Jacob Lorenzo-Morales[2], Nabil Ghedadba[5], Samir Benayache[1], José E. Piñero[2*], Isabel L. Bazzocchi[4*]

[1] Unité de recherche Valorisation des Ressources Naturelles, Molécules Bioactives et Analyses Physicochimique et Biologiques, Université Constantine-1, Route d'Ain El Bey, 25 000 Constantine, Algérie
[2] Instituto Universitario de Enfermedades Tropicales y Salud Pública de Canarias, Universidad de La Laguna, Avda. Astrofısico Fco. Sanchez, S/N, 38203 La Laguna, Tenerife, Canary Islands, Spain
[3] Laboratoire Materiaux-Molecules et Applications, IPEST, University of Carthage, La Marsa, Tunisia
[4] Instituto Universitario de Bio-Orgánica Antonio González, Departamento de Química Orgánica, Universidad de La Laguna, Avenida Astrofísico Francisco Sánchez 2, La Laguna 38206, Tenerife, Canary Islands, Spain
[5] Laboratory of Biotechnology of the Bioactive Molecules and Cellular Physiopathology, Department of Biology, University of Batna 2, Algeria.

Table of Contents

Figure S1: Calibration graphs for total phenolic and flavonoid contents (μg/mL)

Figures S2: ^{1}H NMR spectrum of thymol dervative **1**.

Figure S3. ^{13}C NMR spectrum of thymol dervative **1**.

Figure S4. 2D-COSY NMR spectrum of thymol dervative **1**.

Figure S5. 2D-HMBC NMR spectrum of thymol dervative **1**.

Figure S6. 2D-HSQC NMR spectrum of thymol dervative **1**.

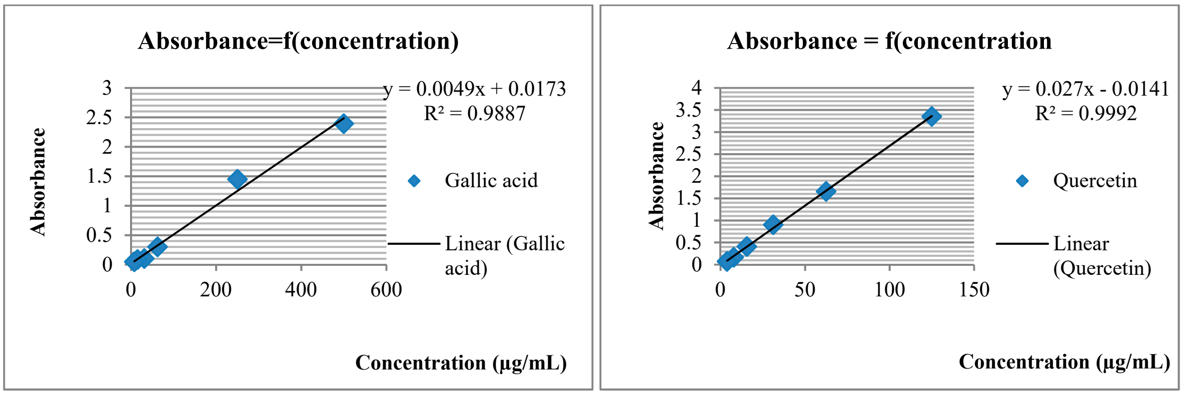

Figure S1. Calibration graphs for total phenolic and flavonoid contents (µg/mL) in *Pulicaria inuloides.*

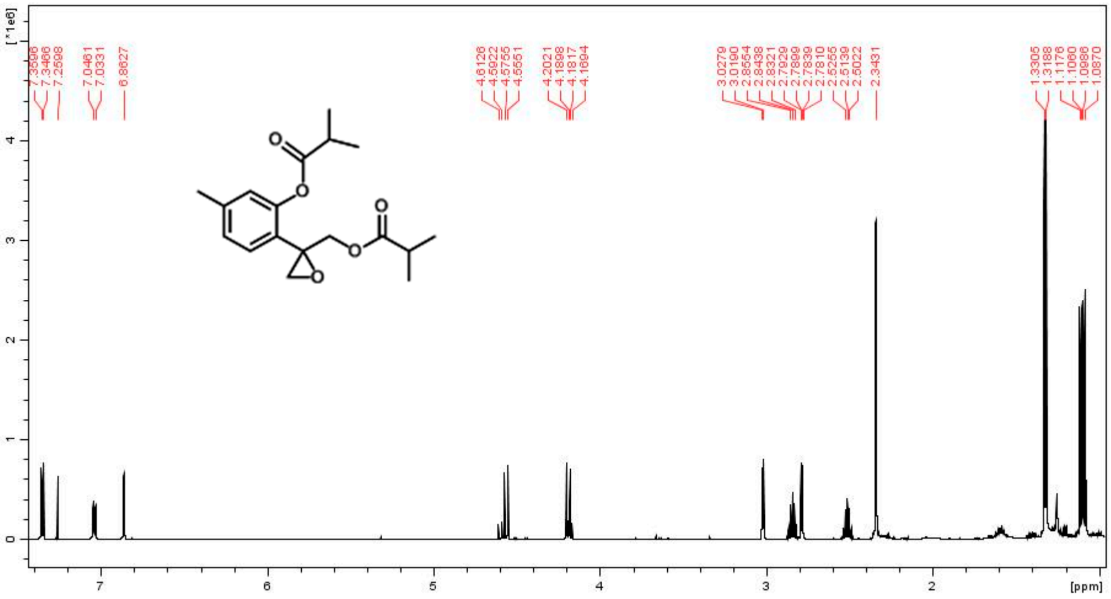

Figure S2. ^{1}H NMR spectrum of **1** in $CDCl_3$ (600 MHz).

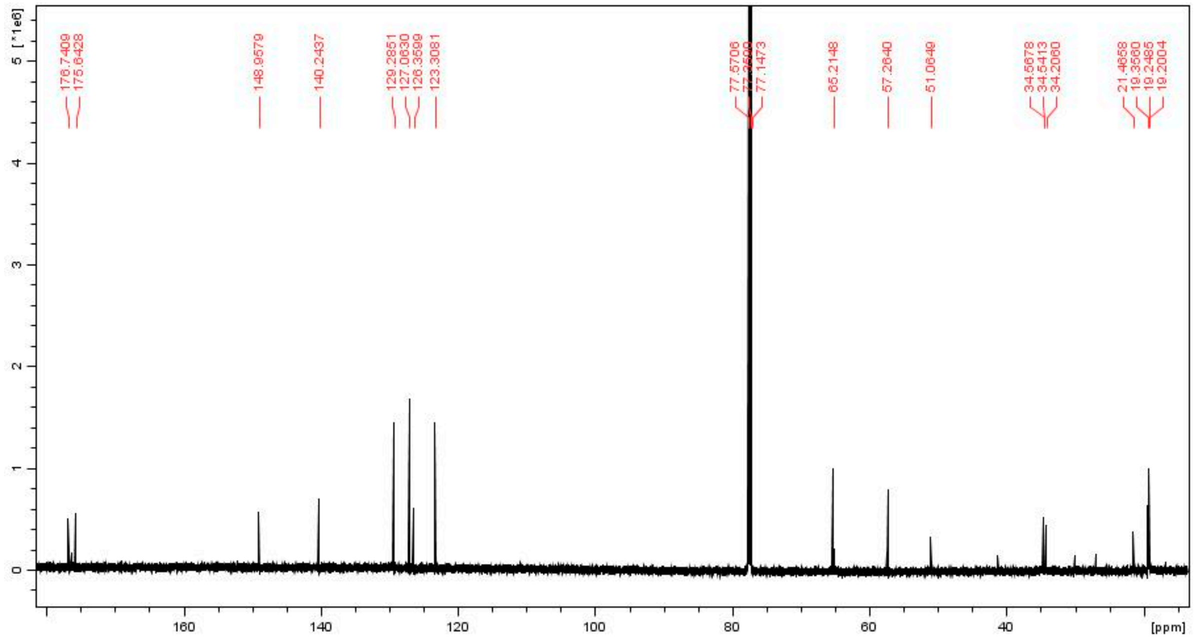

Figure S3. ^{13}C NMR spectrum of **1** in $CDCl_3$ (150 MHz).

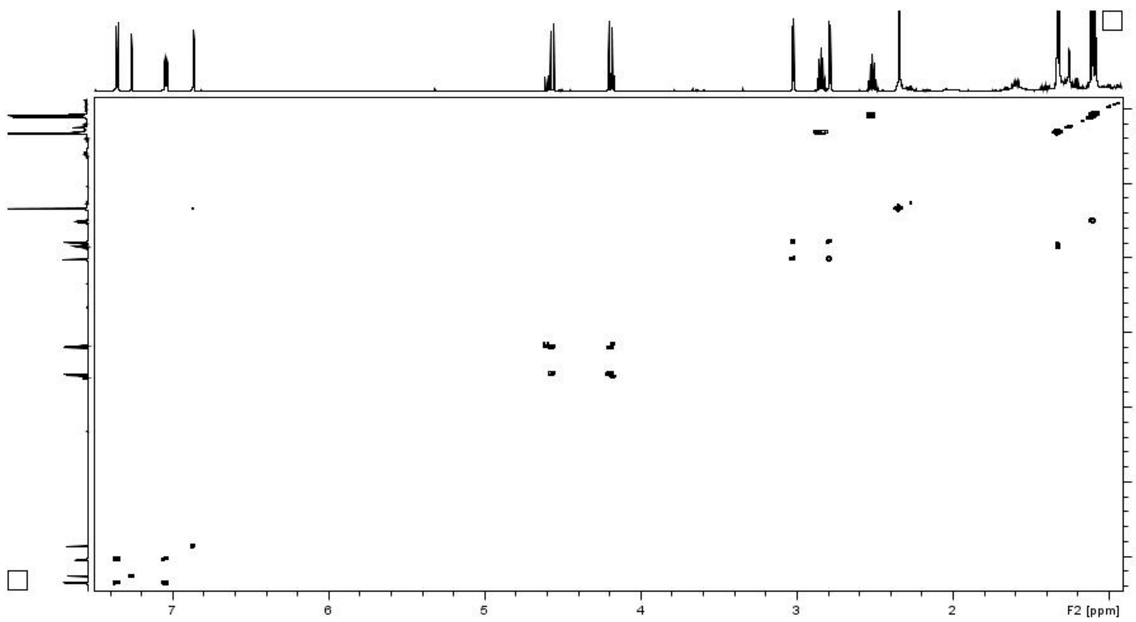

Figure S4. 2D-COSY (^{1}H-^{1}H) NMR spectrum of **1** in $CDCl_3$ (600 MHz).

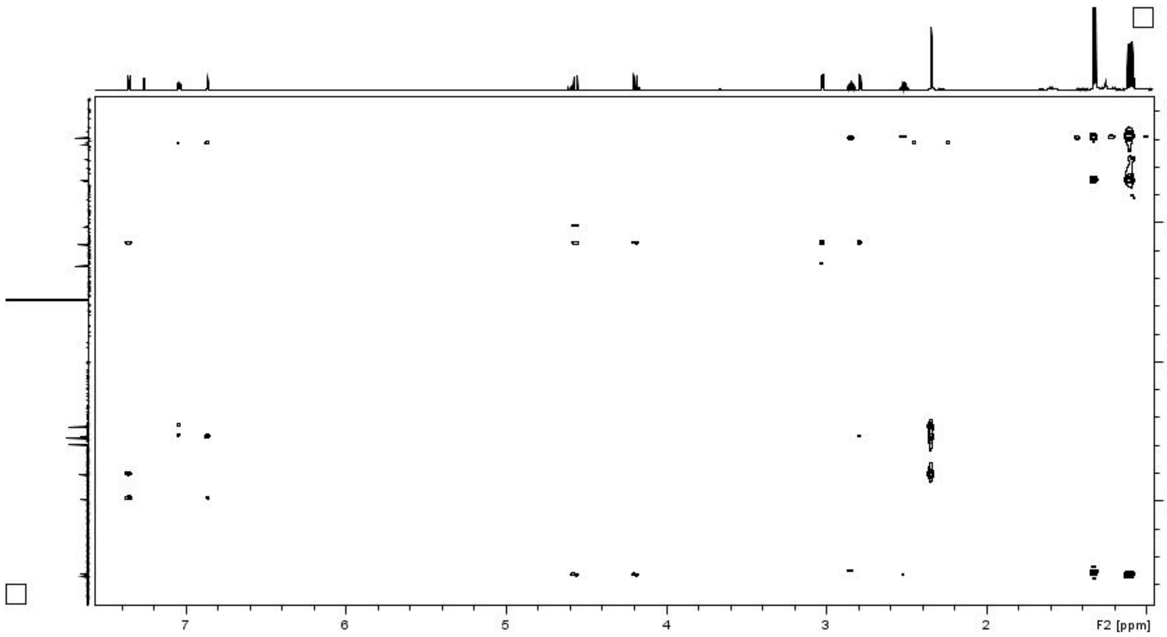

Figure S5. 2D-HMBC (^{1}H-^{13}C) NMR spectrum of **1** in $CDCl_3$ (600 MHz).

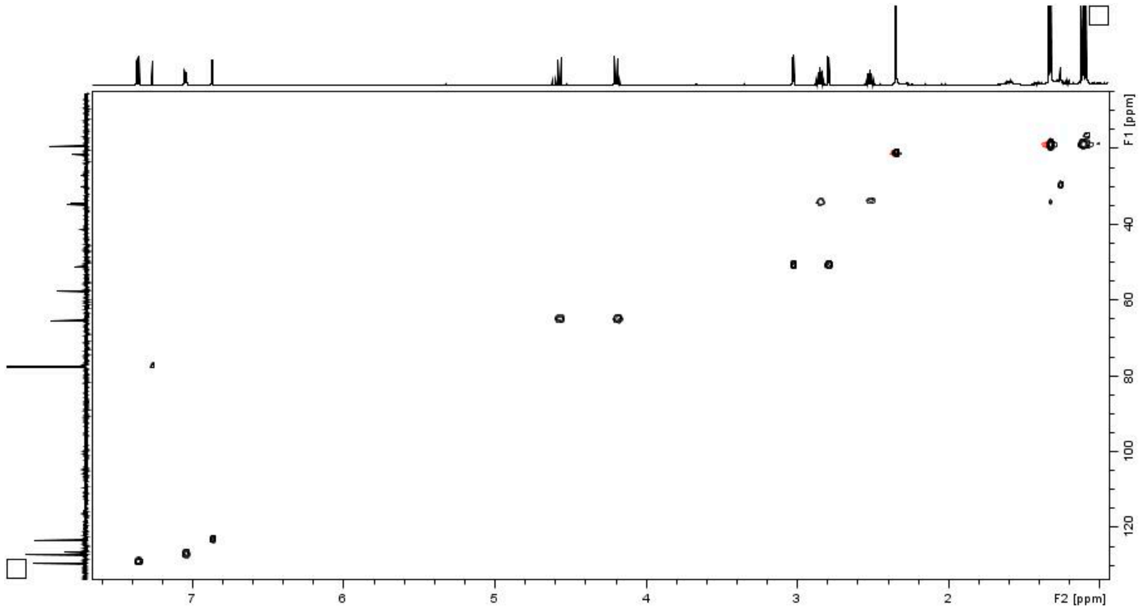

Figure S6. 2D-HSQC (^1H-^{13}C) NMR spectrum of **1** in $CDCl_3$ (600 MHz).
